# Microbial inoculation of seed for improved crop performance: issues and opportunities

**DOI:** 10.1007/s00253-016-7590-9

**Published:** 2016-05-17

**Authors:** Maureen O’Callaghan

**Affiliations:** Lincoln Science Centre, AgResearch Ltd, Private Bag 4749, Christchurch, 8140 New Zealand

**Keywords:** Microbial inoculation, Seed treatment, Rhizosphere, Biopesticide, Formulation, Microbial biomass

## Abstract

There is increasing interest in the use of beneficial microorganisms as alternatives to chemical pesticides and synthetic fertilisers in agricultural production. Application of beneficial microorganisms to seeds is an efficient mechanism for placement of microbial inocula into soil where they will be well positioned to colonise seedling roots and protect against soil-borne diseases and pests. However, despite the long history of inoculation of legume seeds with *Rhizobia* spp. and clear laboratory demonstration of the ability of a wide range of other beneficial microorganisms to improve crop performance, there are still very few commercially available microbial seed inoculants. Seed inoculation techniques used for research purposes are often not feasible at a commercial scale and there are significant technical challenges in maintaining viable microbial inocula on seed throughout commercial seed treatment processes and storage. Further research is needed before the benefits of a wide range of environmentally sensitive potential seed inoculants can be captured for use in agriculture, ecosystem restoration and bioremediation. There is no single solution to the challenge of improving the ability of seed inoculants to establish and function consistently in the field. Development of novel formulations that maintain the viability of both inoculant and seed during storage will result from multidisciplinary research in microbial and seed physiology and adjuvant chemistry.

## Introduction

Global increases in food production achieved in recent decades have required large (15–20 times) increases in the use of synthetic pesticides to control pests, pathogens and weeds of crops (Oerke 2006) but the increasing use of synthetic pesticides is no longer sustainable. Strong consumer pressure has resulted in the withdrawal of many synthetic pesticides, the lowering of maximum residue limits and changes in the regulatory environment that favour more environmentally benign control options. In addition, costs of development and registration of synthetic pesticides have been escalating, leading to significant reductions in development and launch of new chemistries (Glare et al. 2012) further limiting control options available to growers. The search for alternative solutions for agriculture has prompted researchers to take a second look at the range of microorganisms long known to provide benefits to agricultural production and is driving rapid growth in markets for biopesticides (Lehr 2010) and plant growth-promoting microorganisms (Berg 2009).

Regardless of the purpose for which beneficial microorganisms are applied to crops, they must be mass produced and applied in a way that optimises their functionality in the target environment. They have variously been delivered (mainly for research purposes) as liquids (sprays, drenches, root dips) or as dry formulations applied in furrow at time of planting. However, many of these approaches are not feasible on a large scale because of the amount of microbial inoculum needed, particularly in broad acre crops. Application of beneficial microorganisms to seeds is an efficient mechanism for placement of microbial inocula into soil where they will be well positioned to colonise seedling roots or make contact with soil-dwelling invertebrate pests that feed on plant roots. Application of beneficial microorganisms to seeds is not a new idea; the inoculation of legumes with N_2_-fixing bacteria has a long history and underpins the widespread use of legumes of global significance in terms of world food supply (Graham and Vance 2003). However, despite the long history of inoculation of legumes and clear laboratory demonstration of the ability of a wide range of other beneficial microorganisms to improve crop performance, there are still very few commercially available microbial seed inoculants.

**Fig. 1:**
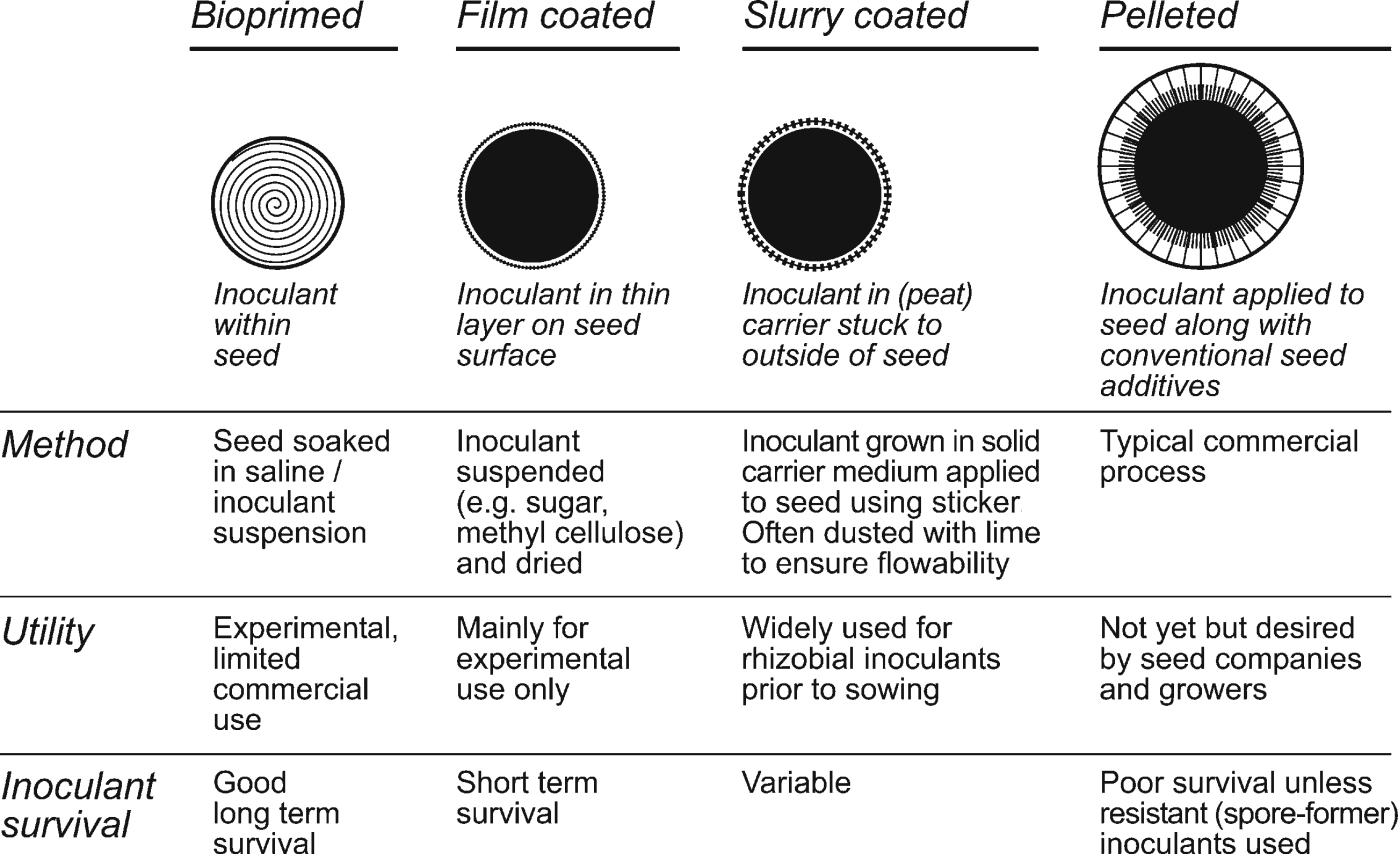
Properties of methods commonly used for microbial inoculation of seed

Most work on microbial seed inoculation involves agrichemical and seed companies and as it can lead to commercial advantage, the techniques and processes used are rarely published and are held as “in house knowledge” or “trade secrets”. Many published studies have described methods for preparation and application of high numbers of microorganisms to seed for research purposes (Table 1), relatively few of which have been scaled for commercial use. The main seed treatment methods are represented in Fig. 1. Bio-priming is the immersion of seeds in a microbial suspension for a pre-determined period, followed by drying of seed (sometimes under vacuum) to prevent onset of germination. Given the effort involved in this process, it is most appropriate for low-medium volume high value crops, such as vegetable seed. Film coating is the application of inoculum as an aqueous cell suspension or in a liquid polymer or adhesive. Materials commonly used include methyl cellulose, vegetable or paraffin oils and polysaccharides. In slurry applications, inoculants formulated as powders or in other carriers (commonly peat) are applied to the outside of seeds using a range of stickers. Film coating has mainly been used experimentally but slurry coating is used extensively for inoculation of legume seeds on farm. Many of the techniques used for research purposes are unsuitable for scale-up to commercial application and there is strong preference from growers and seed companies for a “pre-inoculated seed” that can be purchased and used in the same way as conventionally treated seed. Pre-inoculation of seed presents significant scientific, technological and commercial challenges which must be overcome to meet the rapidly expanding global need for new seed treatments. Demand for biological seed treatment is growing rapidly with some commentators speculating that biological seed treatments have the opportunity to capture as much as 20 % of the global seed treatment market (New Ag International 2015). The fastest growing market for seed treatment in general is in Asia Pacific with the demand for biological seed treatments expected to grow at a compounded annual rate of 9 % from 2014 to 2020 (Mordor Intelligence LLP 2014).

**Table 1:** Microorganisms used for biological seed treatments in research and pre-commercial trials

Function/microorganism	Target	Crop	Seed treatment method	L/GH/F	Reference
Plant disease control					
*Pseudomonas fluorescens*	*Pythium* sp.	Sugarbeet	Bacterial suspension; proprietary formulation	L	Fenton et al. (1992), Moenne-Loccoz et al. (1999)
	*Pythium* sp.	Onion	Patented biopolymer coating	L	O’Callaghan et al. (2006)
*Pseudomonas chlororaphis* ^a^	*Tilletia caries*, *Septoria nodorum*, *Fusarium* spp.	Wheat, barley, peas	Proprietary formulation	F	BioAgri AB (2016)
*Pseudomonas chlororaphis*, *Serratia plymuthica*, *Paenibacillus polmyxa*, *Lysobacter gummosus*	*Didymella bryoniae*	Pumpkin	Bio-priming	F	Furnkranz et al. (2012)
*Serratia plymuthica*	*Verticillium dahliae*	Oilseed rape	Pelleting, film coating, bio-priming	GH	Muller and Berg (2008)
*Microbacterium oleovorans*, *Bacillus amyloliquefaciens*	*Fusarium verticillioides*	Maize	Bacterial suspension	GH, F	Pereira et al. (2007, 2010, 2011)
*Achromobacter xylosoxidans*	*Magnaporthe oryzae*	Rice	Glycerol based formulation	L, GH	Joe et al. (2012)
*Bacillus subtilis* ^a^	*Rhizoctonia*, *Fusarium* spp.	Cotton	Proprietary formulation	F	Brannen and Kenney (1997), Bayer Crop Science (2016a)
*Bacillus amyloliquefaciens*	*Rhizoctonia solani*	Soybean	Bacterial suspension	L, GH	Correa et al. (2009)
	*Fusarium oxysporum*, *Fusarium graminearum*	Soybean	Peat slurry	GH	Zhang et al. (2009)
Bacterial consortia—*Bacillus* spp., *Pseudomonas aeruginosa*, *Streptomyces* sp.	Sunflower necrosis virus disease	Sunflower	Powder and liquid	GH, F	Srinivasan and Mathivanan (2011)
*Paenibacillus polymyxa*	*Fusarium* spp., *Rhizoctonia solani*, *Ralstonia solanacearum*	Sesame	Pelleting	GH, F	Ryu et al. (2006)
*Clonostachys rosea*	*Fusarium culmorum*	Barley	Spore suspension	GH, F	Jensen et al. (2000)
*Clonostachys* sp., *Pseudomonas* spp., *Trichoderma* spp.	Unspecified soil-borne plant pathogens	Carrot, onion	Commercial drum priming	GH, F	Bennett et al. (2009)
*Pseudomonas* sp., *Trichoderma* sp.	*Colletotrichum truncatum*	Soybean	Bio-priming	F	Begum et al. (2010)
*Bacillus subtilis*, *Gliocladium catenulatum*	*Plasmodiophora brassicae*	Canola, crucifer vegetables	Suspension prepared from commercially available products	GH, F	Peng et al. (2011)
*Trichoderma hazianum*	*Plasmophora halstedii*	Sunflower	Conidial suspension	GH, F	Nagaruju et al. (2012)
*Trichoderma* spp.	*Pyrenophora* sp.	Wheat	Conidial suspension	F	Perelló and Dal Bello (2011)
*Streptomyces spororaveus*	*Fusarium udum*	Wheat	Suspension of hyphae and spores	F	Al Sahli and Abdulkhair (2012)
*Pythium oligandrum*	*Rhizoctonia solani*, *Pythium* sp. *Aphanomyces* sp.	Cress, sugar beet	Pelleting and film coating	L	McQuilken et al. (1990)
Weed control					
*Pseudomonas putida*, *Stentotrophomonas maltophilia*, *Enterobacter taylorae*	Weed of wheat crops	Downy brome	Methylcellulose suspension	GH	Mazzola et al. (1995)
*Fusarium oxysporum* f. sp. *strigae*	Root parasitic weed	Witch weed	Film coated	GH	Elzein et al. (2010)
Invertebrate pest control					
*Serratia entomophila*	*Costelytra zealandica*	Carrot, wheat, ryegrass	Patented biopolymer coating	L, GH	Wright et al. (2005); Young et al. (2009, 2010)
*Metarhizium anisopliae*	*Adoryphorus couloni*	Ryegrass	Spores in methylcellulose	L	Rath (1992)
	*Agriotes obscures* (wireworm)	Corn	Conidia applied with corn oil	F	Kabaluk and Ericsson (2007)
*Pseudomonas fluorescens*, *Beauveria bassiana*	Sucking pests and pod borer complex	Pulses	Talc based formulations	F	Soundararajan and Chitra (2011)
*Beauveria bassiana*	Stem borer *Sesamia calamistis*	Maize	Dusting with spore powder	F	Cherry et al. (2004)
	*Helicoverpa zea*	Tomato	Methylcellulose coating	GH	Powell et al. (2009)
*Pasteuria* spp.	Nematode *Rotylenchus reniformis*	Cotton	Spore suspension	GH	Schmidt et al. (2010)
*Bacillus firmus*	Plant parasitic nematodes *Heterodera* spp., *Meloidogyne* spp. *Pratylenchus* spp.	Corn, cotton, sorghum, soybean, sugar beet	Proprietary formulation	F	Bayer Crop Science (2016b)
Improved plant performance					
*Pantoea aglomerans*	Increased rhizosphere soil moisture content	Wheat	Peat inoculant	L	Amellal et al. 1998
*Pseudomonas putida*	Alleviation of drought stress	Sunflower	Talc formulation	L	Sandhya et al. (2009)
*Pseudomonas monteilii*, *Cronobacter dublinensis*, *Bacillus* spp.	Improved yield of essential oil	Sweet basil	Bacterial suspension	F	Singh et al. (2013)
*Bacillus subtilis*	Improved yield—plant biomass and essential oil	Tulsi	Bacterial suspension	GH, F	Tiwari et al. (2010)
*Bacillus* sp.	Improved yield under semi-arid conditions	Cowpea	Charcoal:broth mix	F	Minaxi et al. (2012)
*Bacillus pumilus*, *Micrococcus* spp.	Increased uptake of nickel from soil	Alpine penny-cress (hyperaccumulator)	Methylcellulose suspension	L	Aboudrar et al. (2013)
*Trichoderma hazianum*	Alleviation of salinity stress	Wheat, rice	Bio-priming	L	Rawat et al. (2011, 2012)
	Alleviation of disease pressure and abiotic stress—osmotic, salinity, temperature	Tomato	Conidia coated onto cellulose and encapsulated with tapioca dextran	L	Mastouri et al. (2010)
*Penicillium radicum*, *Penicillium bilaii*	Improved access to soil P	Wheat, medic, lentil	Aqueous spore suspension	L, GH	Wakelin et al. (2007)
*Penicillium bilaii* ^a^	Improved access to soil P	Wheat, canola, corn	Various proprietary formulations, seed treatment applied close to time of sowing	F	Monsanto BioAg (2016)

*L* laboratory (including controlled growth chamber), *GH* glasshouse, *F* field trials

^a^Available as commercial products

Here, published research on the functional groups of seed microbial inoculants is reviewed. The review is not exhaustive, rather the intention is to identify where significant new opportunities may lie for the exploitation of microbial seed inoculants as new solutions for agriculture and beyond. Key constraints limiting commercial development of microbial seed inoculants and research opportunities to address these barriers are identified.

## Seed inoculants for use in agriculture

Microbial inoculants applied as seed treatments deliver microorganisms directly to the plant rhizosphere—the narrow zone of soil that surrounds the roots where plants interact directly with microorganisms (Philippot et al. 2013). It is a zone of intense microbial activity, with growth of plants and microorganisms dependent on reciprocal provision of nutrients and a wide range of other compounds including plant growth regulators and antibiotics. Many beneficial microorganisms of agricultural importance are rhizosphere colonising species, with ability to increase plant growth via a range of mechanisms (Babalola 2010).

### Improved plant nutrient availability

The best known example of seed inoculation is obviously that of legume seeds which aims to maximise yield potential by providing high numbers of viable rhizobia to the rhizosphere to allow rapid colonisation, nodulation and atmospheric nitrogen (N_2_) fixation by a selected inoculant strain (Deaker et al. 2004). For many legume varieties, inoculation with the correct rhizobial partner is essential for crop establishment where the required strain is not present in soil. By the end of the nineteenth century, the practice of mixing “naturally inoculated” soil with seeds became a recommended method of legume inoculation in the USA and the first patent (“Nitragin”) for plant inoculation with *Rhizobium* sp. was registered in 1896 (Bashan 1998). The legume inoculant industry is now well established, a wide range of inoculant products are available around the world and legume inoculation can be considered “the success story” of applied soil microbiology (Catroux et al. 2001).

However, despite this long and successful history, there are still issues with poor survival of some inoculant rhizobia, both on seed and after sowing. Point-of-sale surveys of pre-inoculated legume seeds carried out in Australia have shown that viable numbers of rhizobia on some legume seeds rarely meet the required numerical standards (Gemell et al. 2005; Hartley et al. 2012). Factors affecting inoculant quality and seed survival of rhizobia have been the focus of significant research efforts (reviewed by Deaker et al. 2004) but there remains a clear need for novel solutions to address this well-documented problem.

Plant growth enhancement by some plant associated and soil microorganisms is related to their ability to act as “biofertilisers” by increasing the availability of nutrients in the rhizosphere of plants (Vessey 2003). Concern over security of supply and fluctuating costs of phosphorus fertilisers has resulted in increased interest in microorganisms that aid plant uptake of phosphorus (P) from soil (Richardson and Simpson 2011). A limited number of microbial products that improve plant P uptake are available. JumpStart® (Monsanto BioAg 2016) contains the fungus *Penicillium bilaii* and is recommended for use with wheat and canola, with Canadian wheat growers reporting average yield benefits of ~6 %, with a report of significantly higher yields (up to 66 %) for wheat (Harvey et al. 2009), although other reviews suggest the benefits may be much lower (Karamanos et al. 2010). The fungal inoculant is provided for application to seed close to time of sowing. Numerous biofertiliser products are also under development or currently being marketed in developing countries, particularly in parts of Asia and Latin America. Many of these appear to be produced at low cost primarily for domestic markets.

### Control of plant diseases and plant growth promotion

Treatment of seed with fungicides is often necessary to avoid failure of crop establishment caused by seed or soil-borne plant pathogens. Application to seed of microbial antagonists to soil-borne pathogens is an ideal delivery system as it introduces inoculum to the rhizosphere where plant pathogens such as *Pythium* and *Rhizoctonia* are active, causing seed rots in the spermosphere and seedling damping-off. A range of both bacterial and fungal antagonists have been used experimentally and commercially for this purpose (Butt and Copping 2000; Nelson 2004a; Berg 2009) but they have been used less frequently as seed treatments (McQuilken et al. 1998). When tested experimentally, they have often been used by simply immersing seed in aqueous suspensions of spores/cells (Table 1). Because of the potential shown by *Pseudomonas* isolates for control of intractable soil-borne phytopathogens, there have been several attempts at development of seed coating techniques incorporating *Pseudomonas* spp. with mixed levels of success (O’Callaghan et al. 2006).

*Bacillus* spp. have proven to be ideal candidates for development as stable and efficient biological products because their ability to produce heat-resistant endospores (Errington 2003; Yanez-Mendizabal et al. 2012) which survive the stresses of commercial seed treatment better than nonspore-forming species such as *Pseudomonas* spp. *Bacillus subtilis* was used extensively as a cotton seed treatment in the USA (Brannen and Kenney 1997) and is currently marketed as Kodiak (Bayer Crop Science 2016a). The inoculum can be applied as water-based slurry with other registered seed treatment insecticides and fungicides through standard seed treatment equipment. Other *Bacillus* spp. have been used experimentally as seed treatments; a recent example is improved cowpea seed germination and yield parameters following seed treatment with a *Bacillus* strain with multiple beneficial attributes such as P solubilisation, and anti-fungal and ACC deaminase activity (Minaxi et al. 2012).

The ascomycete fungus *Trichoderma* has also been evaluated frequently as a seed treatment. *Trichoderma* spp. are able to control pathogenic ascomycetes, basidiomycetes and oomycetes and have also shown activity against nematodes (Schuster and Schmoll 2010). *Trichoderma* spp. also enhance plant growth by multiple additional mechanisms including enhancement of plant systemic resistance and increased root proliferation (Schuster and Schmoll 2010). When used as seed treatments, *Trichoderma* spp. have been shown to induce improvements in seed and subsequent crop performance (Harman 2000, 2006). In a rare example of field evaluation of microbial biocontrol seed treatments, *Trichoderma* spp. gave good control of wet root rot of mungbean caused by *Rhizoctonia solani* (Dubey et al. 2011). Many *Trichoderma* products (liquid and granular formulations) are available but to date it has been used infrequently as a seed treatment despite demonstrated potential of this delivery method under greenhouse and field conditions (e.g. Nagaraju et al. 2012).

The benefits of seed treatment with microbial antagonists can extend well beyond plant establishment. For example, treatment of maize seed with *Bacillus amyloliquefaciens* and *Microbacterium oleovorans* reduced populations of *Fusarium verticilliodes* and associated mammalian-active mycotoxins fumonisin B_1_ and B_2_ in grain (Pereira et al. 2007; Pereira et al. 2011). Microbial antagonists can provide plant protection in situations where no chemical treatments are available, such as oilseed rape seed delivery of *Serratia plymuthica* for suppression of pathogen *Verticillium dahlia* (Muller and Berg 2008). In this case efficacy of biocontrol varied with seed treatment method with bio-priming and pelleting of seed providing better plant protection than film coating, indicating the importance of developing an optimal seed treatment process for the microbial agent used.

By far, the greatest research effort has been directed at microbial inoculation of seed for protection against plant pathogens (Table 1). However, despite numerous published records of successful plant disease suppression under experimental conditions, only a handful of microbial seed inoculant products for protection from plant diseases are available commercially. *Pseudomonas chlororaphis* is the active agent in seed treatments Cedomon®, Cerall® and Cedress® (BioAgri AB 2016) available for barley, wheat and peas, respectively. Product information states that *P. chlororaphis* stimulates root growth and early plant establishment and is active against several plant pathogens (e.g. Cerall® is reported to be effective against the seed-borne diseases common wheat bunt including *Tilletia caries*, wheat leaf spot (*Septoria nodorum*) and Fusarium (*Fusarium* spp.) in wheat. The bacterium has been approved for use within the EU and according to the manufacturers, since its launch in 1997, a total of approx. Two million hectares in several countries have been sown with Cedomon® treated seed.

Many of the bacterial genera with biocontrol ability discussed above also act as general plant growth-promoting rhizobacteria (PGPR) using a range of mechanisms, for example through production of phytohormones and siderophores (Lugtenberg and Kamilova 2009; Berg 2009; Babalola 2010; Verbon and Liberman 2016). Microbial inoculation of seed should be an effective and efficient delivery method for PGPR, in particular where plant response depends on colonisation of the rhizosphere. *Burkholderia ambifaria* MCI 7 promoted growth of maize seedlings when applied as a seed treatment, but the same strain reduced plant growth if incorporated directly into soil (Ciccillo et al. 2002). The contrasting effects on plant growth were attributed to different responses of the indigenous microflora, with seed treatment causing a decrease in bacterial diversity in the rhizosphere, while incorporation into soil increased bacterial diversity.

### Control of invertebrate pests

Microbial seed inoculation for protection of crops from damage caused by insect pests, or as a means to deliver biopesticides to soil and plant roots has been attempted infrequently, despite the fact that many soil-borne insect pests are attracted to roots and germinating seeds by volatile compounds released into the soil. Soil dwelling insect pests are difficult to control by any method and seed treatment is useful way of introducing microbial control agents into the root zone where likelihood of contacting root-feeding pests is high. The discovery that the fungal entomopathogen *Metarhizium anisopliae* is an effective coloniser of the plant rhizosphere (Hu and St Leger 2002) suggests application by seed treatment may be appropriate for this agent. *M. anisopliae* has been used experimentally as a seed treatment to protect field corn (*Zea mays*) from yield loss caused by wireworm, *Agriotes obscures* (Kabaluk and Ericsson 2007) and pasture plants from the subterranean pasture scarab redheaded cockchafer, *Adoryphorus couloni* (Rath 1992). Spores of *M. anisopliae* were applied to seeds of a range of pasture species as an alternative to applying a granular formulation directly to soil; seed treatment provided considerable advantages over the grain formulation in terms of ease and costs of storage, handling and transportation.

The nonspore-forming bacterial insect pathogen *Serratia entomophila* has been developed as a commercial microbial control agent (bioshield™) for the New Zealand grass grub, *Costelytra zealandica* and is applied in a granule formulation to established pasture where the soil-dwelling grass grub larvae cause significant economic losses (Jackson 2007). The granular formulation has been developed for broad acre application but targeted application of *S. entomophila* by incorporation into seed treatments may reduce the amount of inoculum required for effective pest control while allowing efficient inoculation into soil and the rhizosphere at the time of sowing, using existing machinery (Townsend et al. 2004). Proof-of-concept for delivery of *S. entomophila* via seed treatment has been demonstrated in a range of crops, including carrots, wheat and ryegrass (Wright et al. 2005; Young et al. 2009, 2010). Significantly, the level of seedling protection afforded by the microbial control agent was similar to that achieved using standard insecticide seed dressings.

### Bionematicides

Nematicidal microorganisms have been used as seed treatments and one of the few examples of a commercially available biological seed treatment is the nematicidal bacterium *Bacillus firmus*—the active ingredient in the product PONCHO/VOTiVO (Bayer Crop Science 2016b). This was originally a stand-alone product, but *B. firmus* is now most commonly used as a seed treatment in combination with the insecticide clothianidin (Poncho). This product is used for control of insect pests and plant parasitic nematodes on a range of crops including corn, cotton, sorghum, soybean and sugar beet (Wilson and Jackson 2013). As a spore-former, *B. firmus* is well suited to withstanding the stresses associated with commercial seed treatment processes with the company claiming 2 years product stability under cool dry conditions. A range of other nematicidal microorganisms are used commercially but delivery is generally via dry granules or granules that can be dissolved and applied as sprays or through irrigation systems. *Pasteuria* spp. are also well recognised as endospore-forming bacterial endoparasites of plant parasitic nematodes. While difficult to mass produce, *Pasteuria* spp. has demonstrated potential as a seed treatment for control of reniform nematodes: population control was comparable to a seed-applied nematicide/insecticide (thiodicarb/imidacloprid) at a seed coating application rate of 1.0 × 10^8^ spores/seed (Schmidt et al. 2010).

### Control of weeds

Seed application could be a useful method for delivery of bioherbicides into cropping systems. This approach requires that the bioherbicide inoculant initially colonises roots emerging from the seed of the crop plant, and then colonises the rhizosphere of adjoining target weeds to a level that inhibits growth of the target weed. Proof-of-concept for this approach has been demonstrated. Downy brome (*Bromus tectorum*) causes significant yield reductions in wheat and rhizobacteria strains (*Pseudomonas putida*, *Stentotrophomonas maltophilia*, *Enterobacter taylori*) capable of inhibiting root elongation or seed germination of downy brome have been identified (Kennedy et al. 1991). Following application to wheat seeds, these bacterial strains successfully colonised the downy brome rhizosphere and reduced the competitive ability of the weed under glasshouse conditions (Mazzola et al. 1995).

### Improved stress tolerance of crops

Drought stress is a key factor limiting crop production in many arid and semi-arid areas of the world and will become an increasing problem under current climate change predictions. There is renewed interest in plant-associated microorganisms capable of ameliorating plant stress via a wide range of mechanisms that span modification of plant hormone levels and production on bacterial exopolysaccarides (Kaushal and Wani 2016). For example, a strain of *Pseudomonas putida* (selected for its ability to survive at low soil moisture potential) colonised the rhizoplane and soil adhering to sunflower roots and increased the percentage of stable soil aggregates. Increased plant biomass and stress tolerance was attributed to plant growth-promoting compounds produced by the bacteria within the biofilm that it produced on the roots of the seedlings, and through production of exopolysaccharide which increased soil aggregation, thereby maintaining higher water potential around the roots (Sandhya et al. 2009). This is a novel approach to increasing stress tolerance in plants but these experiments were conducted under laboratory conditions in sterile soil and proof-of-concept would need to be established in non-sterile soil, which presents a real challenge to colonisation by inoculants. Salt stress is another key abiotic factor limiting crop production. Seed treatment with salinity or drought tolerant isolates of *Trichoderma harzianum* reduced the severity of stress in wheat plants (Rawat et al. 2011; Shukla et al. 2015) under laboratory and greenhouse conditions, respectively. Seed treatment with *T. harzianum* has also improved seed germination under osmotic and salt stress and under suboptimal temperatures in the laboratory (Mastouri et al. 2010) by reducing the damage caused to the stressed plant by accumulation of toxic reactive oxygen species. Priming of Styrian oil pumpkin seed with bacterial endophytic strains (*Serratia plymuthica* and *Lysobacter gummosus*) improved desiccation tolerance of the plants in the field (Furnkranz et al. 2012).

## Challenges in development of microbial inoculant seed treatments

Seeds are treated to enhance profitability so if microbial inoculation of seed is not cost effective or is too time-consuming, it will not be a viable option, regardless of the efficacy of the particular inoculant (Taylor and Harman 1990). For full commercial success and uptake of biological seed treatments, growers will ideally be able to purchase inoculated seed at similar cost and use the seed in the same way they have always purchased pesticide-treated seed (Catroux et al. 2001). Legume seed inoculation by commercial companies can occur on-demand after sale of the seed (custom inoculation) or prior to sale (pre-inoculation) (Deaker et al. 2012). Inoculating seed just prior to sowing removes the need for prolonged shelf-life of the inoculant. However, seed companies and growers have a clear preference for “pre-inoculated” seed that is prepared weeks, and often months in advance of sowing (Catroux et al. 2001; Hartley et al. 2012). Farmers are thus relieved of the inconvenience of inoculation of seed on-farm and seed companies that provide pre-inoculated seed can charge more for this product. For commercial reasons, biological seed treatments should ideally have similar shelf life properties as conventionally treated seed but prolonged survival of most inoculants on seed has not been demonstrated, as discussed below. The success of Cedomon® and related products based on *P. chlororaphis* (BioAgri AB) may be attributable to the claimed compatibility of the microbial treatment with conventional seed treatment and farming practices. The manufacturers claim that Cedomon® treated seed can be stored, transported and handled in the same manner as untreated or conventionally treated seed. In addition to farmer/grower expectations, seed companies have high expectations in terms of cost of production and product shelf-life, expecting that the microbial inoculant will remain viable on stored seed for periods of months-years, in line with seed treated with conventional pesticides. Meeting these considerable expectations is a challenge but opportunities exist at several stages of product development of a biological seed treatment, as discussed below.

### Cost effective production of robust microbial biomass

Seed treatment typically requires less microbial biomass than other application methods. For example, use of *Bacillus firmus* as a seed treatment as opposed to a drench product allows a much reduced load to be applied per unit area (approximately 1000-fold reduction) as the active agent is targeted at the rhizosphere of developing roots (Wilson and Jackson 2013). However, even with these efficiencies, cost-effective methods for production of microbial biomass are still needed. This has been achieved for some biopesticides and biocontrol agents by producing inoculum in media based on waste streams or other inexpensive materials (e.g. Visnovsky et al. 2008; Brar et al. 2005) and various by-products from the food industry (Costa et al. 2001). An economically feasible production system has been identified for *Piriformospora indica*, a root endophytic fungus which exerts plant growth promoting effects on its host plants (Kumar et al. 2011). Manipulation of nutritional and cultural parameters resulted in maximum biomass during the growth phase and subsequent spore yield was optimised by glucose deprivation. It is essential that optimised methods for biomass production methods do not compromise the functionality at the inoculum at the expense of high cell yields at low costs. Fuchs et al. (2000) found that ability of a *Pseudomonas* strain to protect cucumber plants from black root rot increased when it was cultured in “less rich media”, which was attributed to differences in the physiological state of the cells following growth in “rich” and “less rich media”, although no mechanism was determined. This type of study suggests reduced manufacturing costs of biocontrol products can be achieved while simultaneously improving the biocontrol efficacy by replacing rich laboratory media with less rich media.

Understanding of the specific growth parameters and physiology of the inoculant strain is essential to optimise the functionality and stability of the inoculant biomass. While the duration of fermentation (and hence cost) can be reduced by halting the fermentation when maximum cell yield has been achieved at the end of the growth phase, significant benefits in terms of cell survival can be reaped by continuing the fermentation into the stationary phase when the starvation response triggers changes in the regulatory pathways and subsequent metabolism in gram negative bacteria. For the insect pathogenic bacterium *Serratia entomophila*, cells harvested in exponential growth phase survived less than 4 weeks when stored at 4 °C, in contrast to 85–100 % survival when stationary phase cells were harvested (Visnovsky et al. 2008). In one of the very few published studies examining incorporation of microorganisms into commercial seed treatment processes, Moenne-Loccoz et al. (1999) identified that the method used to prepare microbial inoculum was a key factor in maintaining viability of the inoculant *Pseudomonas* strain on seed. Inclusion of additional nutrients in the carrier material in which the inoculant was produced resulted in improved survival of the inoculant on seeds but this did not result in improved control of damping-off disease in a laboratory assay. Efficacy was improved when the inoculant was allowed to “acclimatize” in the carrier material for several days prior to its application to seed.

Stress cross protection has been observed whereby the bacterial starvation response that occurs during stationary phase provides protection against osmotic and temperature stress (Navarro Llorens et al. 2010). These specific microbial stress response mechanisms can be manipulated during cell culture to improve microbial tolerance to adverse environmental conditions. Improving the tolerance of microbial inoculants to desiccation stress in particular is very relevant in consideration of seed treatment where microbes will likely be exposed to rapid (and often heated) drying that is standard in conventional seed treatment. Some microorganisms are known to accumulate intracellular compounds in response to fluctuations in osmotic pressure. Compounds such as trehalose and glycine-betaine are well-known osmoprotectants (Bremer and Kramer 2000; Räsänen et al. 2004) and the role of osmoprotectants in survival of rhizobium is well recognised (Deaker et al. 2004). Osmotically adapted cells can display a greater tolerance to subsequent periods of desiccation. This technique is referred to as anhydrobiotic engineering (Garcia de Castro et al. 2000; Manzanera et al. 2002) and has been shown to improve survival of several bacterial biocontrol agents under dry conditions (Sheen et al. 2013; Pusey and Wend 2012; Sartori et al. 2010). Simple salt treatments to induce mild osmotic stress during culturing of a biocontrol strain of *P. agglomerans* improved ability of cells to tolerate a spray-drying process and maintained their efficacy against postharvest fungal pathogens on fruit (Teixido et al. 2006). Different microorganisms accumulate different osmoprotectants so detailed understanding of the inoculant microbe is required. For example, accumulation of glycine-betaine rather than trehalose was important in improving desiccation tolerance of *S. entomophila* (Sheen et al. 2013). These are techniques that have not yet been used to improve persistence of seed inoculants.

### Formulation and seed treatment processes

Effective formulation is a key factor in development of commercial seed inoculant products based on nonspore-producing microorganisms. The four basic functions of formulation are to stabilise the microbe, aid in delivery of the microbe to the target zone, protect the microbe during seed storage and after planting, and ideally, to enhance the functionality of the microbe in situ (Jones and Burges 1998). Technical aspects of formulation of plant growth-promoting bacteria, including as seed inoculants, have been reviewed recently (Bashan et al. 2014). Essentially seed coating processes have changed little in many decades, with inoculants typically being subjected to a range of processes based on traditional chemical seed treatment equipment and components. A large proportion of seed sold to growers has been commercially treated with proprietary ingredients including pesticides, polymer adhesives, colour pigments or dyes and various powders. Reasonably large amounts of materials can be used for weight build-up and to assist seed-flow through sowing machinery and improve coat integrity. Despite the long history of seed inoculation with rhizobia, poor survival—particularly on pre-inoculated seed—remains a significant problem. Hartley et al. (2012) recommended that each seed treatment ingredient and stage in the seed-coating process be tested for compatibility to determine best practices to promote rhizobial survival on seed. Their study highlighted that even basic factors such as water quality could impact on survival of rhizobia on seed.

In their extensive review, Deaker et al. (2004) noted that while polymeric adhesives such as xanthan gum (or mixtures of gums), methyl cellulose, gum arabic, polyvinyl pyrollidone and polyacrylamide have long been known to improve survival of seed inoculant, studies on seed additives have been ad hoc and little is understood about their mode of action. The protective properties of biopolymers have been attributed to their ability to maintain water activity levels optimal for rhizobia survival, and their ability to limit heat transfer but detailed studies are needed, that take into account complex and multiple interactions between seed and rhizobial cell biology and the physical and chemical properties of seed additives. New formulations are needed that protect or separate the inoculant from materials used routinely for seed treatment and possibly from the seed surface itself.

Perhaps most formulation effort has been targeted at overcoming temperature and desiccation stresses that are encountered in seed treatment processes, where seeds are typically air-dried (often at very high temperatures) for storage purposes. In addition to anhydrobiotic engineering approaches mentioned above, addition of appropriate formulation excipients can improve microbial stability. Compounds such disaccharides and other polyols that enhance stress resistance through accumulation within cells have been utilised as extracellular excipients to protect cells during drying. Hydroxyectoine, a common compatible solute in salt-tolerant bacterial species, improved the survival of *Pseudomonas putida* KT2440 (Manzanera et al. 2002).

There has been renewed interest in bio-encapsulation technologies recently. This work began some time ago with encapsulation of beneficial microorganisms in alginate matrix in beads (e.g. Bashan 1986) and has advanced considerably with development of new materials and technologies. A wide range of encapsulation methods are suitable for microbial inoculants and biological control agents, with the technology having the potential to allow co-formulation of several active ingredients, such as two biological control agents, or a biological agent with a synergistic efficacy enhancing agent such as “attract and kill” capsules (Vemmer and Patel 2013). Given the improved environmental persistence of encapsulated biopesticidal microorganisms, it has been suggested that there is potential to treat seeds with micro-encapsulated inoculants (John et al. 2011). The feasibility of this approach on a commercial scale remains to be determined; the technique requires specialised equipment and involves many sequential steps which will add to costs. However, there is currently considerable commercial interest in this technology as one which may lead to a step-change in microbial seed treatment.

### Shelf-life and storage conditions

The shelf-life of environmentally sensitive microorganisms on seed is another challenging and success-limiting factor in development of new inoculated seed products. Studies documenting the shelf-life (long term viability of the inoculant and its biocontrol activity) of inoculated seed are extremely rare. This may because of reluctance to publish negative results and there is no doubt that maintaining viability of microbes on seed is a significant challenge. Shah-Smith and Burns (1997) reported on survival and biocontrol activity of *Pseudomonas putida* applied to sugar beet seed using two commercial seed treatment processes (film coats sprayed onto re-pelleted seed and incorporation of *P. putida* cells into the pellet material prior to pelleting). Both methods resulted in large initial losses of inoculum at the time of seed treatment but application of high rates ensured the target rate of 6 × 10^7^*P. putida* per seed pellet was achieved. Viable numbers of bacteria remaining on seed after 24 and 52 weeks varied with treatment method and storage temperature but the results were very promising with bacterial viability and biocontrol efficacy of cells applied in pellet material maintained at high levels for 24 weeks when stored at 18–20 °C. Survival in *S. plymuthica* cells applied to oilseed rape seed using three methods that all delivered high initial loadings onto the seed coat (seed pelleting and film coating) and into the seed (bio-priming) differed with storage temperature (Muller and Berg 2008). Survival was improved when seed was stored at 4 °C in comparison with 20 °C following pelleting and filmcoating, but shelf-life was unaffected by temperature when seed was bioprimed. Regardless of which seed treatment method was used, there was a reduction of at least one log after 1-month storage of seed at 4 °C, with up to 4 logs loss for seed stored at 20 °C. The improved survival at lower temperatures was unsurprising and has been reported previously for *P. putida* on sugar beet seeds (Shah-Smith and Burns 1997), and *P. fluorescens* on onion seeds (O’Callaghan et al. 2006). It is well recognised that low temperature extends bacterial survival by reducing metabolic activity, but for inoculated seed to become part of mainstream agriculture, extended survival of inoculants at ambient seed storage conditions is needed.

A recent study has highlighted the complex interactions between formulation methods/excipients and environmental conditions (relative humidity and temperature) on stability of *Trichoderma* spores applied to maize seed (Swaminathan et al. 2016). Spore survival over 6 months varied significantly between five formulations held under different environmental conditions, suggesting that there is not one formulation suitable for storage under all conditions and that changes in relative humidity affect spore survival in some formulations more than others. Further examination of the interactions between the spores, seed coat and excipients will be needed to explain these results but the study raises important issues that must be considered in development of commercial microbial seed inoculant products, including early and clear definition of the conditions likely to be encountered during seed storage in the target market. Suppliers of Cedomon® and related products state that the inoculant can be stored at 4 to 8 °C for up to 8 weeks, or room temperature for up to 3 weeks before use. Interestingly, it is claimed that once the inoculant is applied to seed, treated seed can be stored for up to 1 year but storage temperature is not defined. While benefits of cool storage of inoculated seeds are clear, refrigeration will not be on option in most cases. An exception where cool storage of seed has become standard practice is ryegrass seed carrying proprietary strains of the beneficial *Epichloë* endophytes in New Zealand (Hume et al. 2013). While these seed transmitted endophytes are somewhat different from the inoculants discussed above, it is useful to consider their commercial development. It is well established that *Epichloë* endophytes die during seed storage and all members of the seed supply chain, from seed production companies through to farmers, are encouraged to treat endophyte-infected seed as a perishable high value product. There is significant cost in storing of seed under conditions that maintain both endophyte and seed viability but the benefits in terms of plant production are considered sufficient to warrant this effort.

### Quality control

The need to satisfy the customer of the quality of the product will be important for microbial inoculated seed where the grower may be paying a premium price. Preliminary work to establish optimal seed loadings of inocula will generally be required. For biological control, a minimum loading is required to provide an adequate level of seedling protection but the required loading may vary with the microorganism’s mode of action and ability to colonise the environment. Quality control standards and processes have long been developed for legume inoculant and seed industry (reviewed by Herrmann and Lesueur 2013) and these can serve as a model for new microbial seed inoculants under development. For example, in Australia, inoculants must pass standards based on the number of effective rhizobia in peat that will result in a minimum number of cells per seed after application at the manufacturers recommended rate (Deaker et al. 2004). There are also accepted minimum standards for loadings of rhizobia for various legume seeds (e.g. 10^3^ for small-seeded legumes such as lucerne, up to 10^5^ for larger seeds such as soybean), but these may vary slightly between countries (Lupwayi et al. 2000). Some countries also impose limits of the extent of contamination that is acceptable in the peat cultures. In Canada, Brazil, France and Uruguay, product standards are legislated for, while in Australia, New Zealand and South Africa it is left to the seed companies to voluntarily adhere to the expected standards (Catroux et al. 2001). Similar standards are also in place for temperate forage grass seeds carrying seed transmitted *Epichloë* endophytes, with licence agreements specifying that endophyte must be viable in >70 % of seed to avoid product failure in the field (Hume et al. 2013).

### Product safety and registration

Depending on the intended function of the inoculated seed product, approval by relevant regulatory authorities may be necessary, in particular where the inoculant is acting as a biopesticide. Regulatory procedures for pre-market assessment of safety vary between jurisdictions (Kabaluk et al. 2010 but data requirements for registration should be considered early in the product development pathway. The risks associated with microbes used to target pests and diseases relate to their toxicity, infectivity, pathogenicity and displacement of non-target organisms (Cuddeford and Kabaluk 2010) but many of these risks are minimised when inocula are used as a seed treatment as opposed to being applied to the broader environment, for example as a spray. Relatively low amounts of inocula are used in seed treatments and there is typically reduced exposure of non-target organisms, including to the growers and farmers using the seed treatments. However, the challenges associated with product registration should not be underestimated; the process can be expensive and time consuming. It is important that potential pathogenic microorganisms are excluded early in product development and there are several screening methods available to rapid first evaluation (Berg 2009).

### Consistent field performance

Despite frequent demonstration of efficacy in laboratory and glasshouse experiments over many decades, there are relatively few published studies confirming field efficacy of biological seed treatments and most have been published in recent years (Table 1). Biological control agents in general have often proved to be less effective and more variable than chemical control options under field conditions. This may be due to initial failure of establishment on the seed or in the rhizosphere due to unfavourable environmental conditions or biotic factors such as competition with resident microbial populations, or a host of other reasons. In many cases, promising results in laboratory and greenhouse studies have not translated to field efficacy. Screening methods used early in the strain selection process may not sufficiently model field conditions and good performance in vitro does allow prediction of field performance. However, the frequency of papers reporting field trial results appears to be increasing and this trend is likely to continue with the growing interest and expanding market for biological seed treat treatments.

Comparison of field performance of microbial seed treatments against conventional chemical treatments is essential and builds confidence in seed inoculation products. For example, performance of soybean seed bioprimed with *Pseudomonas aeruginosa* was measured in two field trials (carried out over two seasons within one year). Significant increases in seed germination and reductions in both pre- and post-emergence damping off caused by *Colletotrichum truncatum* were reported with the level of disease control achieved with an inoculant strain comparable to that achieved using the standard chemical fungicide treatment Benlate® (Begum et al. 2010). Similarly, favourable field performance of biological seed treatments (combinations of endophytic *Paenibacillus plymyxa*, *Serratia plymuthica*, *Pseudomonas chloraphis* and *Lysobacter gummosus*) in comparison with chemical seed treatment was also reported following three field trials conducted over 2 years with Styrian oil pumpkins (Furnkranz et al. 2012). Germination rates were equivalent to those achieved using chemical treatments, and increased emergence rates and decreased disease incidence were also observed with some inoculants. In addition to increased yield, seed inoculation can also improve quality of the crop. For example, while essential oil yield was improved by 45–56 % in *Ocimum basilicum* (sweet basil) following inoculation of seeds with bioinoculant *Pseudomonas monteilii*, *Chromobacter dublinensis*, and *Bacillus* spp. (Singh et al. 2013), one inoculant strain also gave improved oil quality by yielding measurably higher amounts of oil components responsible for basil aroma. This type of field study is encouraging but extensive testing at multiple sites (covering effects of soil types, climatic conditions etc) will be necessary to build confidence in seed biological products and few products have been tested extensively in this way. Product information on the biofertiliser product Jumpstart® (based on *Penicillium bilaii*) suggests that multiple field trials (several hundred) have been undertaken to support product claims; improved dry matter production in canola (average of 6 %) was reported from 163 farmer-conducted trials using seed pre-treated with Jumpstart (Monsanto BioAg 2016). Little data has been published but it is clear that there has been significant investment in field evaluation of this product.

### Compatibility of microbes with grower practice

Most biological seed treatments will likely be used in combination with conventional farming and crop management practice, materials and equipment which may include the use of agrichemicals. Thus, it will be important to test the efficacy of biological seed treatments and their associated microorganisms under conditions likely to be encountered on farm. Testing the compatibility of seed inoculants with groups of agrichemicals commonly used as seed dressings is an important starting point as seed dressings with pesticides have been shown to adversely affect the structure and function of beneficial soil microorganisms, in particular for rhizobia inoculants where pesticides have been shown to severely reduce production of legumes (e.g. Fox et al. 2007).

Neonicotinoids, for example imidocloprid, are broad-spectrum systemic insecticides that are frequently applied to seeds prior to planting to protect seedlings from early-season root and leaf-feeding on many crops including cotton, corn, cereals, sugar beet and oilseed rape (Elbert et al. 2008). Restrictions on the use of imidocloprid and other neonicotinoids as sprays are in place in many countries but they remain a key component of seed dressings for control of soil pests. The use of neonicotinoid seed treatments may negatively impact beneficial leaf-feeding insects that are important biological and natural control agents in integrated pest management programs (Moser and Obrycki 2009). The prospect of deregistration of this pesticide in future is driving interest in biological seed treatments but while it remains in use, there is a need to ensure that potential microbial candidates for use in seed treatments are compatible with this and other components of commercial seed treatments. Little is known about the impact of imidocloprid on non-target microorganisms and testing of seed inoculants for compatibility with this chemical would be useful if the two are to be used in combination on seed; recent studies have reported some effects of imidacloprid on soil microbial community function, in particular on N cycling bacteria (Cyon and Piotrowska-Seget 2015).

## Research needs and opportunities

Despite extensive research on utility of numerous microorganisms beneficial to agriculture, few are used as seed treatments. For example, in a recent review of beneficial microorganisms for increasing productivity in cotton cropping systems, not one of the biofertiliser and biocontrol products currently marketed for use in cotton production was a seed treatment (Pereg and McMillan 2015). There is no single solution to the challenge of improving the ability of seed inoculants to carry out their required functions under a wider array of conditions and with minimal variability in performance but given the potential benefits to be reaped from this approach, further research and development is needed.

### Novel biomass production, formulation and seed treatment processes

Production and formulation are closely linked processes that underpin the development of commercially viable microbial seed treatments. To produce a large, stable and efficacious biomass for each microbial agent for use in formulation, intensive study is needed to identify the optimal production method (solid state or fermentation) and medium constituents, production parameters (e.g. temperature, oxygen transfer, time and method of harvest) and post-harvest treatment of cell biomass (Hynes and Boyetchko 2006). Fundamental understanding of the empirical observations on the beneficial effects of peat (Casteriano et al. 2013) or osmoprotectants (McIntyre et al. 2007) provides new research leads needed to make a step-change in production of microbial seed inoculants.

In addition to improvements in production of robust cell biomass, progress in commercialisation of microbial seed treatments is dependent on development of novel formulation technologies that balance the biological requirements of the seed (e.g. conditions needed to maintain seed viability and good germination rates) against the specific conditions needed for maintenance of the inoculum, which may differ from that of the seed. Research effort into formulation of microorganisms has intensified with the recent company mergers and acquisition of “biologicals” companies by agrochemical companies. Doubtless significant research effort is now going into development of improved formulations that will support seed delivery of beneficial microorganisms. While most of this research remains commercially sensitive, it is clear that some new approaches with potential application to microbes are being explored, for example novel microencapsulation techniques. Whether or not these techniques can be adapted to seed treatment remains to be seen. The use of several of these technologies in combination may be needed to overcome inherent incompatibility in conditions required for optimal seed vs microbial survival. For example, bacteria dried with osmoprotectants remained viable when encapsulated in plastic coatings applied to maize seeds (Manzanera et al. 2004). Whether this multi-step process could be scaled up economically is uncertain, but it provides a useful proof-of-concept.

It is clear that many current commercial seed treatment practices (e.g. rapid drying at high temperatures) will present a significant challenge to maintaining viability of microorganisms on seed. While some improvements in inoculant survival will be achieved through formulation, it will be necessary for seed treatment companies to consider other production and storage options to ensure product quality. Slow drying of seed has often been shown to improve survival of inoculants on seed (e.g. Hartley et al. 2012). Drying of seed treated with *P. fluorescens* (20 vs 3 h) enhanced bacterial survival (Moenne-Loccoz et al. 1999) but this prolonged drying period would not fit comfortably with current high throughput commercial practices. These tensions and challenges would be best addressed through collaborative effort between seed treatment technicians and researchers with expertise in microbial formulation.

### Seed and rhizosphere biology

The natural microflora on seed has been regarded as “contamination” by those inoculating seed and this natural contamination has been shown to impact on inoculant survival, at least for rhizobia. Hence, many researchers have pre-sterilised seeds prior to treatment with the desired inoculant but this is not practical on a commercial scale. Little is known about the early fate of inoculants in the spermosphere, which is the short-lived, rapidly changing and microbiologically dynamic zone of soil around a germinating seed (Nelson 2004b). The vast majority of seed exudates (sugars, sugar alcohols, amino acids, organic acids, and various volatiles and enzymes) are released in the early stages of germination and the proliferation of the diverse microbial community present on the surface of seeds is stimulated. Greater understanding of the characteristics and dynamics of seed-associated microflora and seed inoculants in the spermosphere will provide insights into strategies for more effective seed inoculation, for example spermosphere colonising traits can vary among strains of the same inoculant species (Simon et al. 2001) and inoculants may differ in their ability to colonise different plants (Roberts et al. 1992). The success of microbial seed treatments depends not only on formulations that ensure survival of the inoculant during seed treatment and storage, but also on its ability to multiply in the spermosphere, colonise the root and in some cases the surrounding soil, where it then perform its required function. The interactions of seed applied microorganisms with other components of the soil biota have not been well characterised but will have a profound effect on the ability of the inoculant to establish in soil and on roots. For example, soil nematodes played a role in root colonisation and transport of wheat seed applied *Pseudomonas fluorescens* (Knox et al. 2004). Research into the fate of seed inoculants in the rhizosphere has rarely been undertaken but utilisation of new molecular techniques to track and monitor activity of inoculants at the plant-soil interface will provide new knowledge that could be used to guide development of improved selection systems for high-performing inoculant strains and formulations tailored to optimise the biological activity of these.

### Co-inoculants and microbial consortia

There is evidence that co-inoculation with multiple microorganisms can improve plant yield compared to the use of a single inoculant. Use of multiple biocontrol agents with differing targets and modes of action may assist in overcoming some of the variability observed in field trials, where pest/pathogen complexes may be active (Guetsky et al. 2001). Co-inoculation may also broaden the environmental range over which the seed treatment is effective, for example by supplying strains active at a range of different soil temperatures. Single and dual inoculations of field-grown wheat with two isolates of P-solubilising microorganisms (*Pseudomonas* sp. and *Aspergillus awamori*) resulted in significant gains in yield and P uptake (Babana and Antoun 2006). Nodulation by rhizobia was shown to be improved when rhizobia were combined with plant growth promoting rhizobacteria quite some time ago (e.g. Grimes and Mount 1984) and many more recent studies have shown that simultaneous infection with specific PGPR increases nodulation and growth in a wide variety of legumes (Dileep Kumar et al. 2001; Tilak et al. 2006; Sáncheza et al. 2014). Positive plant growth responses as a result of co-inoculation of P-solubilising microorganisms with N_2_-fixers have also been reported in leguminous crops (Rosas et al. 2006; Valverde et al. 2006). Tag Team® (Monsanto BioAg) for use in pulse crops combines a rhizobial inoculant with the P-solubilising fungus *P. bilaii* used in JumpStart®. There may be increased cost associated with production of multiple inoculant strains but where clear benefits can be shown, this cost is justified. Compatibility between the co-inoculants and their modes of action is essential.

### Plant endophytes

There is increasing interest in the role of microbial endophytes in plant function and performance (Hardoim et al. 2015) and their potential as seed inoculants. They live within plant tissues for at least part of their life cycle without causing symptoms of disease and can provide the host plant with a number of advantages including increased protection from pests and diseases and improved tolerance to environmental conditions such as drought stress. Significant increases in rice yield were achieved by seed inoculation with the endophytic bacterium *Achromobacter xylosoxidans*, which suppressed symptoms of rice blast disease by stimulating production of plant defense-related enzymes (Joe et al. 2012). Similarly, inoculation of seeds with a wide range of putative endophtytic bacterial isolates improved shoot dry weights in maize seedlings (Montanez et al. 2012). Endophytic colonisation of a range of crop plants by the insect pathogenic fungus *Beauveria bassiana* has been demonstrated (Cherry et al. 2004; Posada and Vega 2006). The potential utility of seed inoculation with endophytic *B. bassiana* strains for large scale control of the bark beetles (*Hylastes* and *Hylurgus* spp.) in New Zealand pine forests (*Pinus radiata*) was examined, as conventional spray or broadcast application techniques were not feasible because of the high costs of treating large forested areas (Brownbridge et al. 2012). Seed coating facilitated inoculation of *B. bassiana* into *P. radiata*, but at lower levels than had been achieved for other plants (Quesada-Moraga et al. 2006). In a more recent study, inoculation of cotton seed with conidia of *B. bassiana* and with *Purpureocillium lilacinum* facilitated establishment of these plant pathogens and resulted in reduced survival of cotton bollworm (*Helicoverpa zea*) and increased growth of cotton (Costillo Lopez and Sword 2015). Reduced survival of larvae was not caused by mycosis of the insects and further research is needed to determine the mode of action of pest suppression and the mechanism underpinning the observed plant growth enhancement but these promising results demonstrate the potential of this approach, especially given the successful suppression of cotton aphid (*Aphis gossypii*) populations in field trials over two seasons following a simple seed treatment protocol (Castillo Lopez et al. 2014). To date, there has been limited research on the ability of insect pathogenic fungi that establish within the plant following seed treatment to maintain themselves in plant tissue through ongoing growth and reproduction of the plant. Ideally, the inoculant would become established throughout the plant and be vertically transmitted with the seed (as is the case with *Epichloë* endophytes in ryegrass) but this has yet to be demonstrated for the endophytic seed inoculants described above. However, even in the absence of vertical transmission, there should be significant opportunities in terms of selection of improved inoculants from within the endomicrobiome of plants.

### Integrated crop protection systems

The integration of biological and chemical control systems holds promise as it may increase the reliability of crop protection under conditions that are not optimal for performance of the biological control agent alone. There is particular interest in exploiting synergies between microorganisms and so-called soft chemistries such as insect growth regulators to achieve more rapid knockdown of pests while initiating an epizootic of disease in the pest population, to achieve more enduring pest suppression. In trials where the biological and chemical treatments are highly effective alone, additive effects may not be detected in short-term experiments. However, it could be expected that the use of the combined treatment may have advantages over the use of either method alone in the field with the insect pathogens being able to establish and recycle within the pest population resulting in pest suppression for several years, unlike the relatively short-term control achieved using chemical pesticides. As discussed in the section on bionematicides above, one of the current commercial biological seed treatments is PONCHO/VOTiVO™ mix (Bayer Crop Science 2016b) which combines the activity of the insecticide clothinanidin with the nematicidal bacterium *Bacillus firmus* (Wilson and Jackson 2013). Complimentary short-term effects were reported when *Pseudomonas aureofaciens* was used in combination with the fungicide imazalil in a sweet corn seed treatment—there was effective control of *Pythium* seed rot and increased seedling vigour (Mathre et al. 1995).

As discussed above, the key issue in combining conventional and biological approaches in seed treatment will be compatibility between the actives. In vitro testing of direct toxicity of pesticides likely to be used in close association with seed inoculants is a useful starting point. For example, Ahemad and Khan (2012) tested varying rates of compounds from four fungicide families against a plant growth promoting and P-solubilising strain of *Pseudomonas putida*. The ability of the strain to produce plant growth promoting compounds was impaired at the recommended and two and three times the recommended rate of the fungicides in a concentration-dependent manner. Efforts have been made to select for pesticide resistant strains of beneficial microorganisms, to overcome inhibition of pesticides on beneficial microorganisms including rhizobia (Ahemad and Khan 2011).

## Conclusion

In their review of progress and research needs to achieve microbial inoculation to seeds, McQuilken et al. (1998) wrote “opinions differ on how widely microbes will be commercially delivered in the future and how long it will take to happen. As yet, few products have been marketed successfully”. It is true to say that little has changed with respect to the numbers of products on the market, but what has changed is the impending withdrawal of chemical pesticides commonly used as seed treatments. In conjunction with this, there are increasing public and regulatory pressures to provide benign alternatives to chemical pesticides and fertilisers. These factors are driving increasing commercial investment in development of biological solutions for agriculture including development of microbial seed treatments (e.g. BASF 2016). Realisation of the benefits of microbial inoculation of seed will depend on greater collaborative research efforts between researchers and industry. Success likely depends on a multidisciplinary approach incorporating several fields of knowledge including microbial, seed and rhizosphere ecology (to understand the multiple complex interactions between the microbial seed inoculant, seed, target pest seed and the abiotic and biotic factors at play); microbial physiology (to optimise production and formulation variables); seed physiology (to provide insight into the seed surface chemistry and biology that may influence microbial survival and activity and ensure that seed quality and performance is unimpaired); and chemistry (to select compatible adjuvants that support extended shelf-life which includes both maintenance of viable inoculants and the functionality/efficacy of the inoculant).

Research progress in this area has broad applications beyond sustainable agriculture. For example, a future role for biological seed treatments in bioremediation has been demonstrated with seed applied microorganisms facilitating improved uptake of heavy metals from soil (Ma et al. 2011; Aboudrar et al. 2013). While the critical role of rhizosphere microbial communities in plant production is well recognised, there is growing potential to manipulate these rhizosphere communities to provide further solutions for nature conservation, development of bio-energy crops and mitigation of climate change (Philippot et al. 2013). Effective microbial inoculation of seeds will underpin progress in these areas.
